# Methods for Improving the Variance Estimator of the Kaplan–Meier Survival Function, When There Is No, Moderate and Heavy Censoring-Applied in Oncological Datasets

**DOI:** 10.3389/fpubh.2022.793648

**Published:** 2022-05-26

**Authors:** Habib Nawaz Khan, Qamruz Zaman, Fatima Azmi, Gulap Shahzada, Mihajlo Jakovljevic

**Affiliations:** ^1^Department of Statistics, University of Science and Technology (UST), Bannu, Pakistan; ^2^Department of Statistics, University of Peshawar, Peshawar, Pakistan; ^3^Department of Mathematics and Sciences, College of Humanities and Sciences, Prince Sultan University, Riyadh, Saudi Arabia; ^4^Institute of Education and Research, University of Science and Technology, Bannu, Pakistan; ^5^Institute of Advanced Manufacturing Technologies, Peter the Great St. Petersburg Polytechnic University, St. Petersburg, Russia; ^6^Institute of Comparative Economic Studies, Hosei University, Tokyo, Japan; ^7^Department of Global Health Economics and Policy, University of Kragujevac, Kragujevac, Serbia

**Keywords:** Kaplan-Meier, survival analysis, adjusted hybrid variance estimators, leukemia, thalassaemia, cancer, oncology

## Abstract

In case of heavy and even moderate censoring, a common problem with the Greenwood and Peto variance estimators of the Kaplan–Meier survival function is that they can underestimate the true variance in the left and right tails of the survival distribution. Here, we introduce a variance estimator for the Kaplan–Meier survival function by assigning weight greater than zero to the censored observation. On the basis of this weight, a modification of the Kaplan–Meier survival function and its variance is proposed. An advantage of this approach is that it gives non-parametric estimates at each point whether the event occurred or not. The performance of the variance of this new method is compared with the Greenwood, Peto, regular, and adjusted hybrid variance estimators. Several combinations of these methods with the new method are examined and compared on three datasets, such as leukemia clinical trial data, thalassaemia data as well as cancer data. Thalassaemia is an inherited blood disease, very common in Pakistan, where our data are derived from.

## Introduction

The main focus in survival analysis is usually based on the observed time until some event occurs. The event of interest may for instance be death or disease in medical science or component breakage in the area of engineering. In survival analysis, three approaches are used for fitting the models (1) Parametric approach, (2) Non-parametric approach, and (3) Semi-parametric approach. Each approach is based on different techniques. In this paper, we describe the work on the familiar non-parametric approach, i.e., the Kaplan–Meier. In case of no covariate, the Kaplan–Meier survival function may be constructed to estimate the true survival curve.

The Kaplan–Meier is based on the different assumptions and if the population from which the data for a Kaplan–Meier estimation are sampled violate one or more of the Kaplan–Meier assumptions, the results of the analysis may be incorrect or misleading (1). Apart from this, there are some other cases which may also affect the results. For example, in case of small censoring the Kaplan–Meier yields good results but heavy censoring and small sample size may affect the reliability of the Kaplan–Meier estimates. In their paper, Kaplan and Meier (2) adopted the Greenwood's variance estimator. In case of small sample size, the Greenwood's estimator underestimates the true variance. Instead of the Greenwood's formula (3), one may use the Peto et al. (4) variance estimator for the Kaplan–Meier survival function. But it also underestimates the true variance in case of small sample size and heavy censoring.

To overcome these difficulties, one may use a simple regular and adjusted hybrid variance estimator (5) for the Kaplan–Meier survival function. It gives the variance at the censoring time as well as the shrunken estimates of survival function greater than zero and variance even if the last observation is event, i.e., the curve of shrunken never touches the 0 and 1 probability. Just like the Kaplan–Meier, it has its own boundaries. But the problem of this method is that it always gives the variance larger than the Greenwood's and Peto's variance, even if we have no censoring time and it is only useful for a very small sample size.

With these concerns, we present a variance estimator for the Kaplan–Meier. The beginning of any study is very important and if there is heavy censoring at this stage before any event occurred, neither the estimates nor the variance can be found at this point. This means that if we apply the Kaplan–Meier survival function or the shrunken Survival function at these stages, they give constant values, e.g., KM gives 1 until the first event and similarly Shrunken KM also yield some constant value depending upon the *n*. At the first event, these values suddenly change which shows that these ignored censored observations carry some weight. This affects the estimates as well as the standard error (*SE*) too. In addition, when plotting the curves it is unclear, whether the Kaplan–Meier survival function or shrunken Kaplan–Meier function was applied, because both methods are unable to give estimates at the censoring time.

In the calculation of Kaplan–Meier, we considered the number of events and the number of persons at risk. The number of persons at risk also includes the censored observations, but gives no importance to it. This is incorrect because if a censoring occurred in stage one it would not be counted in the next stage and that would affect the number of persons at risk and ultimately also the estimates as well as the variance too. The number of persons at risk at each stage has its own importance.

Keeping these facts in mind here, we introduce a weight, which we assign to those numbers of persons at risk at time (*t*_*i*_) which include the censored observations. On the basis of this weight, we propose a modified cumulative hazard function, the plot of which gives more detailed information in case of heavy censoring in the initial stage as well as later stages.

## Methods

### The Modified Kaplan–Meier Estimator of the True Survival Curve

Let the survival times *T*_1_, *T*_2_,…, *T*_n_ be independently identically distributed and let *C*_1_, *C*_2_, …, *C*_*n*_ be independently identically distributed censoring times. The observed random variables *Y*_*i*_ = min {*T*_*i*_, *C*_*i*_} and σ_*i*_ = *I* {*I*_*i*_ ≤ *C*_*i*_}, is the indicator function, denotes whether the survival time is censored or uncensored.

Then the Kaplan–Meier product limit estimator is defined by


(1) $$\begin{array}{l} \overset{\hat{\;}}{S_{KM}}\;(i)=\underset{i}{\prod }\left(\frac{r_{i}-d_{i}}{r_{i}}\right) \end{array}$$

Where *r*_*i*_ denotes the number of persons at risk at time *T*_*i*_ and *d*_*i*_ denotes the number of events.

In order to develop a procedure here, we considered the total number of observations, number of persons at risk, and serial number, which play the important role in the calculation of probabilities.

The *n* denotes the number of subjects in the sample and *r*_*i*_ denotes the number of persons at risk at time *t*_*i*_. *K* is the serial number in reverse order of those times at which censoring occurred; e.g., if the first observation is censored then it has the highest serial number and if the second observation is censored it has the second highest number and so on. Then the weight is defined by


(2) $$\begin{array}{l} w_{i}=\left(1-\frac{\left(k*I_{ci}\right)}{\left(n*r_{i}\right)}\right) \end{array}$$

Where *I*_*ci*_ is an indicator function and

*I*_*ci*_ = 1 if the observation is censored

= 0 otherwise.

The Kaplan–Meier estimator of the survivor function for any value of *t* in the interval from *t*_(i)_ to *t*_(i+1)_ can be written as


(3) $$\begin{array}{l} \overset{\hat{\;}}{S_{KM}}\;(i)=\underset{i}{\prod }\left(\frac{r_{i}-d_{i}}{r_{i}}\right) \end{array}$$

Where


$$\begin{array}{l} p_{i}=\left(\frac{r_{i}-d_{i}}{r_{i}}\right) \end{array}$$

We assign the weight to it and say


$$\begin{array}{l} p_{i}^{*}=W_{i}*P_{i} \end{array}$$

Therefore,


$$\begin{array}{l} {\overset{\hat{\;}}{S}}_{w\left(i\right)}=p_{1}^{*}p_{2}^{*}\ldots P_{i}^{*}\\ {\overset{\hat{\;}}{S}}_{w\left(i\right)}=\left(W_{1}\;*\;P_{1}\right)\;*\;\left(W_{2}*P_{2}\right)\;*\;\left(W_{3}*P_{3}\right)\ldots \left(W_{i}*P_{i}\right)\\ {\overset{\hat{\;}}{S}}_{W\left(i\right)}={\overset{\hat{\;}}{S}}_{W\left(i-1\right)}\;*\;P_{i}^{*} \end{array}$$

So


(4) $$\begin{array}{l} {\overset{\hat{\;}}{S}}_{W}\;(i)=\overset{n}{\underset{i=1}{\prod }}\left[\left[\frac{r_{i}-d_{i}}{r_{i}}\right]*\left[1-\frac{k*I_{ci}}{n*r_{i}}\right]\right] \end{array}$$

With the variance, the proof of which is given in the appendix.


(5) $$\begin{array}{l} \mathrm{var}\left\{{\overset{\hat{\;}}{S}}_{W}\;(i)\right\}\approx {\left[{\overset{\hat{\;}}{S}}_{W}\;(i)\right]}^{2}\\ \;\left\{\underset{i}{\sum }\left(\frac{\left(k*I_{ci}\;(r_{i}-d_{i})+n*r_{i}*d_{i}\right)}{r_{i}^{2}n\left(r_{i}-d_{i}\right)}\left[1-\frac{k*I_{ci}}{n*r_{i}}\right]\right)\right\} \end{array}$$

The *SE* is


(6) $$\begin{array}{l} SE\left({\overset{\hat{\;}}{S}}_{W}\;(i)\right)\approx \left[{\overset{\hat{\;}}{S}}_{W}\;(i)\right]\\ \;{\left\{\underset{i}{\sum }\left(\frac{\left(k*I_{ci}\;(r_{i}-d_{i})+n*r_{i}*d_{i}\right)}{r_{i}^{2}n\left(r_{i}-d_{i}\right)}\left[1-\frac{k*I_{ci}}{n*r_{i}}\right]\right)\right\}}^{{\;}^{1}{/}_{2}} \end{array}$$

### Effect of $\overset{\wedge }{S_{KM}}\;\text{AND}\;\overset{\wedge }{S_{KM^{*}}}$ on the Weighted Variance

In order to select the best method under different circumstances, i.e., to select the appropriate method, which is suitable in all circumstances, whether there are no censoring, moderate, or heavy censoring, we took the different combinations of $\overset{\wedge }{S_{KM}},\overset{\wedge }{S_{KM^{*}}}$, Greenwood's and Peto's variances with the new method. The combinations are given below:

We replace $\overset{\wedge }{S_{W}}$ by the $\overset{\wedge }{S_{KM}}$ and $\overset{\wedge }{S_{KM^{*}}}$, so the variances and *SEs* are


(7) $$\begin{array}{l} \mathrm{var}\left\{\overset{\wedge }{S_{KM}}\;(i)\right\}\approx {\left[\overset{\wedge }{S_{KM}}\;(i)\right]}^{2}\\ \;\{\underset{i}{\sum }\left(\frac{\left(k*I_{ci}\;(r_{i}-d_{i})+n*r_{i}*d_{i}\right)}{r_{i}^{2}n\left(r_{i}-d_{i}\right)}\left[1-\frac{k*I_{ci}}{n*r_{i}}\right]\right)\} \end{array}$$

(8) $$\begin{array}{l} SE\left(\overset{\;}{{\overset{\hat{\;}}{S}}_{KM}\;(i)}\right)\approx \left[\overset{\wedge }{S_{KM}}\;(i)\right]\\ \;{\left\{\underset{i}{\sum }\left(\frac{\left(k*I_{ci}\;(r_{i}-d_{i})+n*r_{i}*d_{i}\right)}{r_{i}^{2}n\left(r_{i}-d_{i}\right)}\left[1-\frac{k*I_{ci}}{n*r_{i}}\right]\right)\right\}}^{{\;}^{1}{/}_{2}} \end{array}$$

(9) $$\begin{array}{l} \mathrm{var}\left\{\overset{\wedge }{S_{KM^{*}}}\;(i)\right\}\approx {\left[\overset{\wedge }{S_{KM^{*}}}\;(i)\right]}^{2}\\ \;\{\underset{i}{\sum }\left(\frac{\left(k*I_{ci}\;(r_{i}-d_{i})+n*r_{i}*d_{i}\right)}{r_{i}^{2}n\left(r_{i}-d_{i}\right)}\left[1-\frac{k*I_{ci}}{n*r_{i}}\right]\right)\} \end{array}$$

(10) $$\begin{array}{l} SE\left(\overset{\;}{{\overset{\hat{\;}}{S}}_{KM^{*}}\;(i)}\right)\approx \left[\overset{\wedge }{S_{KM^{*}}}\;(i)\right]\\ \;{\left\{\underset{i}{\sum }\left(\frac{\left(k*I_{ci}\;(r_{i}-d_{i})+n*r_{i}*d_{i}\right)}{r_{i}^{2}n\left(r_{i}-d_{i}\right)}\left[1-\frac{k*I_{ci}}{n*r_{i}}\right]\right)\right\}}^{{\;}^{1}{/}_{2}} \end{array}$$

In Greenwood's and Peto's formula, we replace $\overset{\wedge }{S_{KM}\;}\;by\;{\overset{\hat{\;}}{S}}_{W}$, to check the effect of each and every combination.


(11) $$\begin{array}{l} SE\left(\overset{\;}{{\overset{\hat{\;}}{S}}_{G}\;(i)}\right)\approx \left[\overset{\wedge }{S_{w}}\;(i)\right]{\left\{\underset{i}{\sum }\left(\frac{d_{i}}{r_{i}\;(r_{i}-d_{i})}\right)\right\}}^{{\;}^{1}{/}_{2}} \end{array}$$

(12) $$\begin{array}{l} SE\left(\overset{\;}{{\overset{\hat{\;}}{S}}_{p}\;(i)}\right)\approx {\overset{\wedge }{S}}_{w}\;(i)\sqrt{{\;}^{\left[1-\overset{\wedge }{S_{w}\;(i)}\right]}{/}_{r_{i}}} \end{array}$$

### Case of Events Only or No Censoring Times

Here, we studied the behavior of these methods, if the dataset was free from censoring, then what will these methods behave, we studied here.

If the dataset comprises all the events, then all ${w_{i}}^{\prime }s=1$ for *i* = 1,2,..,n

And thus


$$\begin{array}{l} {\overset{\hat{\;}}{S}}_{W}\;(i)=\overset{n}{\underset{i=1}{\prod }}\left[\frac{r_{i}-d_{i}}{r_{i}}\right]=\overset{\;}{{\overset{\hat{\;}}{S}}_{KM}\;(i)}. \end{array}$$

Moreover, in case of events only included in a dataset, $\overset{\;}{{\overset{\hat{\;}}{S}}_{KM}\left(i\right)}$ is just the complement of the empirical distribution function (6), given by


$$\begin{array}{l} \overset{\;}{\overset{\wedge }{S}\left(i\right)=\frac{Number\;of\;individuals\;with\;survival\;times\;\geq \;i}{Number\;of\;individuals\;in\;the\;data\;set}} \end{array}$$

So our function is also equal to the empirical survival function. And the variance is


(13) $$\begin{array}{l} \mathrm{var}\left\{{\overset{\hat{\;}}{S}}_{W}\;(i)\right\}\approx {\left[{\overset{\hat{\;}}{S}}_{W}\;(i)\right]}^{2}\\ \;\{\underset{i}{\sum }\left(\frac{\left(k*I_{ci}\;(r_{i}-d_{i})+n*r_{i}*d_{i}\right)}{r_{i}^{2}n\left(r_{i}-d_{i}\right)}\left[1-\frac{k*I_{ci}}{n*r_{i}}\right]\right)\} \end{array}$$

Since

*W*_*i*_ = 1 *and I*_*ci*_ = 0, in case of all events so


(14) $$\begin{array}{l} Var\left\{{\overset{\hat{\;}}{S}}_{W}\;(i)\right\}\approx {\left[\overset{\hat{\;}}{S\left(i\right)}\right]}^{2}\overset{\;}{\underset{i}{\sum }}\frac{d_{i}}{r_{i}\;(r_{i}-d_{i})} \end{array}$$

Which is reduced to the Greenwood's formula, but in case of events only, i.e., if there are no censored survival times, then *r*_*i*_ − *d*_*i*_ = *r*_*i*+1_ (7), and so


$$\begin{array}{l} \overset{n}{\underset{i}{\sum }}\frac{d_{i}}{r_{i}\;(r_{i}-d_{i})}=\overset{n}{\underset{i}{\sum }}\left(\frac{1}{r_{i+1}}-\frac{1}{r_{i}}\right)=\frac{r_{1}-r_{n+1}}{r_{1}r_{n+1}},\\ which\;can\;be\;written\;as\;\frac{1-\overset{\wedge }{S\left(i\right)}}{r_{1}\overset{\wedge }{S\left(i\right)}},\\ where\;\overset{\;}{\overset{\hat{\;}}{S}\left(i\right){=}^{r_{n+1}}{/}_{r_{n}}} \end{array}$$

Hence, the variance is reduced to the binomial variance, i.e.,


(15) $$\begin{array}{l} Var\left\{{\overset{\wedge }{S}}_{W}\;(i)\right\}=Var\left\{\overset{\wedge }{S_{KM}}\;(i)\right\}=Var\left\{\overset{\hat{\;}}{S}\left(i\right)\right\}\\ \;\approx \frac{\overset{\;}{\overset{\hat{\;}}{S}\left(i\right)\left(1-\overset{\wedge }{S\left(i\right)}\right)}}{r_{1}} \end{array}$$

This means that the results will be same whether the Weighted, Greenwood, or Binomial formulas are used.

### Relationship Among Variances in Case of No Censoring Times

As we know that in case of no censoring, our method and Greenwood's method are just reduced to binomial variance, we are now going to establish the relation among the binomial variance ($Var\left\{\overset{\wedge }{S}\left(i\right)\right\}$) and the variances of Peto and shrunken function. As


(16) $$\begin{array}{l} Var\left\{\overset{\hat{\;}}{S}\left(i\right)\right\}\approx \frac{\overset{\;}{\overset{\hat{\;}}{S}\left(i\right)\left(1-\overset{\wedge }{S\left(i\right)}\right)}}{r_{1}}\\ Var\left\{\overset{\;}{\overset{\wedge }{S}\left(i\right)}\right\}=\frac{{\left(\overset{\wedge }{S}\overset{\;}{\left(i\right)}\right)}^{2}\left(1-\overset{\;}{\overset{\wedge }{S}\left(i\right)}\right)r_{i}}{r_{1}r_{i}\overset{\hat{\;}}{S\left(i\right)}}\\ Var\left\{\overset{\;}{\overset{\wedge }{S}\left(i\right)}\right\}=\frac{r_{i}Var_{p}\left\{\overset{\wedge }{S\left(i\right)}\right\}}{r_{1}\overset{\;}{\overset{\wedge }{S}\left(i\right)}}\\ Var_{p}\left\{\overset{\wedge }{S\left(i\right)}\right\}=\frac{r_{1}\overset{\wedge }{S\left(i\right)}Var\left\{\overset{\wedge }{S\left(i\right)}\right\}}{r_{i}} \end{array}$$

Since

$\frac{r_{1}\overset{\;}{\overset{\wedge }{S}\left(i\right)}}{r_{i}}\leq 1$, so $Var_{p}\left\{\overset{\wedge }{S\left(i\right)}\right\}\leq Var\left\{\overset{\wedge }{S\left(i\right)}\right\}$

With the help of this formula, the variance of Peto can be calculated.

In case of no censoring, the adjusted hybrid variance formula is


(17) $$\begin{array}{l} Var\left\{{\overset{\wedge }{S}}_{H^{*}}\;(i)\right\}\approx \frac{\overset{\;}{\overset{\hat{\;}}{S_{KM^{*}}}\;(i)\left(1-\overset{\wedge }{S_{KM^{*}}\;(i)}\right)}}{r_{1}} \end{array}$$

Where


$$\begin{array}{l} {\overset{\wedge }{S}}_{KM^{*}}\;(i)=\left(1-\frac{1}{r_{1}}\right)\overset{\;}{\overset{\wedge }{S}\left(i\right)+\frac{1}{2r_{1}}} \end{array}$$

By simplifying the variance, we get


(18) $$\begin{array}{l} Var\left\{{\overset{\wedge }{S}}_{H^{*}}\;(i)\right\}=\frac{\left(2r_{1}-1\right)}{4\overset{3}{r_{1}}}+\frac{\overset{\;}{\overset{\wedge }{S}\left(i\right)\left(1-\overset{\wedge }{S}\left(i\right)\right)}}{r_{1}}\frac{{\left(r_{1}-1\right)}^{2}}{r_{1}^{2}} \end{array}$$

(19) $$\begin{array}{l} Var\left\{{\overset{\wedge }{S}}_{H^{*}}\;(i)\right\}=\frac{\left(2r_{1}-1\right)}{4\overset{3}{r_{1}}}+Var\left\{\overset{\wedge }{S\left(i\right)}\right\}\frac{{\left(r_{1}-1\right)}^{2}}{r_{1}^{2}} \end{array}$$

Which implies that


(20) $$\begin{array}{l} Var\left\{{\overset{\wedge }{S}}_{H^{*}}\;(i)\right\}\geq Var\left\{\overset{\wedge }{S\left(i\right)}\right\} \end{array}$$

whether there are no, moderate, or heavy censoring.

So from eqs (1) and (2), we conclude that in case of no censoring time


$$\begin{array}{l} Var\left\{\overset{\hat{\;}}{S_{p}\;(i)}\right\}\leq Var\left\{{\overset{\wedge }{S}}_{\;}\;(i)\right\}\leq Var\left\{\overset{\;}{{\overset{\hat{\;}}{S}}_{H^{*}}\;(i)}\right\} \end{array}$$

(check it) something wrong peto is greater than greenwood.

This will be verified with the help of examples.

### Notations

Since we use different combinations for the variances, we denote these in the following way:

S_kM_ for the Kaplan–Meier survival function

S_KM*_ for the shrunken Kaplan–Meier survival function

S_w_ for weighted Kaplan–Meier survival function

SE_G_ for the greenwood *SE*

SE_P_ for the Peto's *SE*

SE_H_ for regular *SE*

SE_H*_ for adjusted hybrid *SE*

SE_W_ for weighted *SE*

SE_KMW_ for *SE* of weighted method by taking the Kaplan–Meier survival probabilities.

SE_KM*W_ for *SE* of weighted method by taking the shrunken Kaplan–Meier survival probabilities.

SE_WG_ Greenwood's *SE* by taking the weighted survival probabilities.

SE_WP_ Peto's *SE* by taking the weighted survival probabilities.

SE_WH_ Adjusted hybrid *SE* by taking the weighted survival probabilities.

*r*_*i*_ denotes the number of persons at risk at time *T*_*i*_ and *d*_*i*_ denotes the number of events.

*I*_*ci*_ is an indicator function.

## Simulation Study

### Method of Simulations

For the simulation, we used the Borkowf method. A simulation study is designed to compare the average of the ten *SEs* with the simulated *SD* at three quartiles. We generated the survival times {*t*_*i*_} from the Weibull's and Gamma distribution with shape parameters <1 (we took 0.5), = 1, and > 1 (we took 1.5), the hazard function decreases, constant, and increases over time, respectively. Uniform distribution is selected for the censoring time {*C*_*i*_} with density *f* (*t*/*b*) = 1/*b* for 0 ≤ *t* ≤ *b* and 0 otherwise. Where different values of b's are taken for different censoring percentages.

For each underlying distribution, i.e., Weibull and Gamma, we generated 500 datasets of survival times as well as the censoring times from uniform distribution with sample sizes 10, 15, 20, 25, 30, 35, 40, 45, 50, and 100. Then from the survival times and censoring times, we obtained the observed survival times {y_i_} and event indicator variables {σ_i_} (see Table 1).

**Table 1:** Simulated *SD*s and mean standard errors (*SE*s) for ten variance estimators at three quartiles, with data generated from the Weibull and Gamma distribution with different shape parameters and with uniform censoring (based on b), based on 500 simulated trials.

				**Simulated SDs**	**Greenwood SEs**	**Peto SEs**	**Reg. Hybrid SEs**	**Adj. Hybrid SEs**	**Weighted SEs**	**SE_*KMW*_**	SE_KM*W_	**SE_WG_**	**SE_WP_**	**SE_WH_**
**Distribution n π_Gb_**				**Q_1_ Q_2_ Q_3_**	**Q_1_ Q_2_ Q_3_**	**Q_1_ Q_2_ Q_3_**	**Q_1_ Q_2_ Q_3_**	**Q_1_ Q_2_ Q_3_**	**Q_1_ Q_2_ Q_3_**	**Q_1_ Q_2_ Q_3_**	**Q_1_ Q_2_ Q_3_**	**Q_1_ Q_2_ Q_3_**	**Q_1_ Q_2_ Q_3_**	**Q_1_ Q_2_ Q_3_**
Weibull	10	0.55	1.3	0.084 0.201 0.170	0.036 0.134 0.160	0.027 0.116 0.149	0.034 0.130 0.160	0.045 0.138 0.162	0.089 0.155 0.137	0.038 0.146 0.187	0.037 0.151 0.178	0.079 0.124 0.100	0.074 0.145 0.121	0.107 0.191 0.155
shape = 1	15	0.66	0.9	0.057 0.214 0.198	0.014 0.100 0.132	0.011 0.087 0.128	0.012 0.090 0.132	0.018 0.094 0.135	0.062 0.129 0.112	0.014 0.107 0.164	0.014 0.109 0.160	0.054 0.093 0.076	0.056 0.124 0.101	0.078 0.164 0.128
		0.56	1.3	0.086 0.182 0.087	0.037 0.133 0.133	0.029 0.123 0.130	0.032 0.128 0.132	0.035 0.130 0.135	0.091 0.129 0.112	0.038 0.148 0.160	0.036 0.150 0.155	0.081 0.103 0.084	0.081 0.125 0.104	0.108 0.147 0.121
		0.44	1.9	0.101 0.110 0.041	0.072 0.145 0.126	0.058 0.137 0.123	0.065 0.142 0.126	0.075 0.159 0.145	0.103 0.129 0.112	0.075 0.159 0.145	0.073 0.161 0.141	0.095 0.111 0.093	0.091 0.124 0.107	0.114 0.147 0.121
	20	0.42	2.1	0.100 0.071 0.021	0.077 0.129 0.106	0.077 0.129 0.106	0.065 0.127 0.105	0.068 0.128 0.109	0.098 0.112 0.099	0.080 0.143 0.123	0.080 0.143 0.121	0.090 0.096 0.083	0.091 0.110 0.095	0.109 0.126 0.106
		0.56	1.3	0.089 0.172 0.084	0.036 0.122 0.112	0.029 0.119 0.112	0.030 0.117 0.112	0.031 0.119 0.115	0.085 0.112 0.098	0.013 0.106 0.149	0.035 0.139 0.134	0.074 0.089 0.074	0.079 0.111 0.093	0.098 0.133 0.108
	25	0.56	1.3	0.086 0.157 0.042	0.033 0.116 0.099	0.027 0.117 0.101	0.026 0.111 0.099	0.029 0.112 0.101	0.078 0.101 0.087	0.034 0.132 0.123	0.034 0.133 0.119	0.068 0.080 0.067	0.075 0.101 0.083	0.089 0.118 0.095
	30	0.42	2.1	0.097 0.056 0.012	0.075 0.104 0.085	0.069 0.107 0.086	0.064 0.104 0.085	0.065 0.105 0.087	0.083 0.092 0.080	0.078 0.116 0.100	0.080 0.112 0.097	0.076 0.079 0.067	0.083 0.092 0.078	0.091 0.102 0.084
	35	0.42	2.1	0.094 0.016 0.010	0.075 0.097 0.079	0.071 0.101 0.080	0.064 0.098 0.079	0.064 0.098 0.080	0.078 0.085 0.074	0.078 0.109 0.092	0.080 0.109 0.090	0.071 0.074 0.062	0.079 0.085 0.072	0.085 0.094 0.078
		0.76	0.6	0.030 0.198 0.249	0.003 0.046 0.085	0.002 0.045 0.091	0.002 0.039 0.085	0.003 0.039 0.090	0.031 0.087 0.073	0.003 0.052 0.118	0.003 0.052 0.118	0.026 0.055 0.044	0.031 0.088 0.068	0.037 0.112 0.083
	40	0.35	2.6	0.063 0.011 0.008	0.080 0.087 0.072	0.080 0.090 0.073	0.071 0.088 0.072	0.072 0.088 0.074	0.072 0.080 0.069	0.084 0.097 0.083	0.087 0.097 0.081	0.067 0.071 0.060	0.073 0.080 0.068	0.078 0.086 0.073
		0.79	0.5	0.017 0.168 0.294	0.001 0.030 0.078	0.001 0.029 0.085	0.001 0.023 0.078	0.002 0.025 0.083	0.023 0.081 0.068	0.001 0.034 0.111	0.001 0.034 0.111	0.020 0.048 0.040	0.024 0.083 0.064	0.029 0.108 0.077
	45	0.35	2.6	0.060 0.010 0.008	0.077 0.082 0.068	0.078 0.085 0.069	0.069 0.083 0.068	0.069 0.083 0.069	0.068 0.075 0.065	0.081 0.091 0.078	0.083 0.091 0.076	0.063 0.067 0.056	0.070 0.075 0.064	0.074 0.082 0.068
	50	0.39	2.3	0.076 0.024 0.007	0.072 0.079 0.065	0.073 0.084 0.066	0.063 0.081 0.065	0.062 0.081 0.066	0.066 0.072 0.062	0.076 0.090 0.075	0.076 0.090 0.074	0.060 0.062 0.052	0.068 0.072 0.061	0.072 0.078 0.065
		0.66	0.9	0.063 0.217 0.020	0.013 0.075 0.072	0.011 0.083 0.077	0.009 0.068 0.072	0.009 0.067 0.075	0.050 0.072 0.062	0.014 0.088 0.097	0.014 0.088 0.096	0.042 0.053 0.043	0.054 0.074 0.060	0.057 0.087 0.067
		0.76	0.6	0.026 0.189 0.178	0.003 0.038 0.076	0.002 0.038 0.085	0.002 0.030 0.076	0.002 0.030 0.080	0.027 0.073 0.061	0.003 0.043 0.110	0.002 0.043 0.108	0.023 0.047 0.038	0.028 0.075 0.059	0.031 0.093 0.069
		0.79	0.5	0.017 0.148 0.276	0.001 0.020 0.072	0.001 0.020 0.081	0.001 0.016 0.072	0.001 0.016 0.077	0.016 0.073 0.061	0.001 0.023 0.103	0.001 0.023 0.104	0.014 0.044 0.035	0.017 0.075 0.058	0.019 0.096 0.069
	100	0.39	2.3	0.038 0.004 0.003	0.057 0.056 0.045	0.065 0.060 0.046	0.050 0.057 0.045	0.050 0.057 0.046	0.047 0.051 0.044	0.061 0.063 0.053	0.061 0.063 0.053	0.042 0.044 0.037	0.050 0.051 0.043	0.051 0.055 0.046
		0.4	2.2	0.049 0.004 0.003	0.057 0.056 0.045	0.065 0.060 0.047	0.050 0.057 0.045	0.050 0.057 0.046	0.047 0.051 0.044	0.061 0.064 0.053	0.061 0.064 0.053	0.042 0.044 0.037	0.051 0.052 0.043	0.052 0.055 0.046
Shape < 1 = 0.5														
	10	0.56	0.8	0.072 0.221 0.213	0.022 0.103 0.143	0.017 0.092 0.134	0.020 0.100 0.143	0.021 0.105 0.147	0.070 0.155 0.139	0.023 0.111 0.159	0.019 0.114 0.155	0.053 0.143 0.125	0.053 0.143 0.125	0.073 0.187 0.150
		0.55	0.9	0.076 0.222 0.197	0.026 0.107 0.145	0.020 0.096 0.136	0.024 0.105 0.145	0.027 0.109 0.148	0.070 0.200 0.139	0.027 0.115 0.160	0.023 0.118 0.155	0.055 0.128 0.120	0.053 0.144 0.126	0.072 0.184 0.149
	15	0.6	0.6	0.053 0.218 0.169	0.012 0.075 0.120	0.009 0.071 0.118	0.010 0.072 0.120	0.010 0.074 0.124	0.043 0.129 0.113	0.012 0.083 0.138	0.011 0.084 0.135	0.039 0.123 0.105	0.043 0.129 0.113	0.049 0.153 0.120
		0.55	0.9	0.070 0.212 0.107	0.020 0.103 0.121	0.016 0.097 0.117	0.017 0.099 0.121	0.017 0.099 0.124	0.063 0.201 0.113	0.020 0.113 0.134	0.018 0.115 0.131	0.055 0.106 0.100	0.056 0.123 0.106	0.069 0.148 0.119
		0.5	1.3	0.078 0.195 0.102	0.025 0.114 0.120	0.021 0.109 0.117	0.022 0.111 0.120	0.024 0.112 0.123	0.073 0.129 0.113	0.026 0.125 0.132	0.025 0.126 0.129	0.064 0.110 0.103	0.065 0.124 0.107	0.079 0.145 0.117
		0.44	1.65	0.095 0.151 0.100	0.044 0.128 0.118	0.036 0.123 0.114	0.039 0.126 0.118	0.037 0.127 0.121	0.090 0.129 0.113	0.045 0.139 0.126	0.042 0.140 0.124	0.080 0.114 0.105	0.080 0.123 0.107	0.096 0.141 0.116
		0.37	2.5	0.101 0.105 0.013	0.058 0.136 0.117	0.048 0.130 0.113	0.053 0.135 0.117	0.055 0.135 0.119	0.101 0.128 0.113	0.060 0.145 0.122	0.062 0.147 0.120	0.089 0.118 0.107	0.088 0.123 0.108	0.105 0.138 0.116
	20	0.65	0.45	0.036 0.212 0.209	0.005 0.061 0.102	0.004 0.058 0.101	0.004 0.055 0.102	0.005 0.057 0.105	0.034 0.113 0.108	0.005 0.068 0.121	0.005 0.069 0.119	0.029 0.083 0.080	0.032 0.109 0.100	0.039 0.136 0.106
		0.6	0.6	0.043 0.226 0.149	0.007 0.069 0.103	0.006 0.069 0.103	0.006 0.066 0.103	0.007 0.068 0.106	0.042 0.112 0.100	0.007 0.078 0.121	0.006 0.079 0.118	0.035 0.087 0.083	0.039 0.109 0.093	0.048 0.132 0.105
		0.49	1.3	0.087 0.172 0.059	0.029 0.109 0.101	0.025 0.110 0.100	0.024 0.108 0.101	0.023 0.108 0.104	0.071 0.112 0.099	0.030 0.121 0.111	0.028 0.122 0.109	0.063 0.096 0.089	0.067 0.109 0.094	0.077 0.124 0.102
		0.44	1.7	0.093 0.145 0.010	0.035 0.112 0.099	0.030 0.112 0.097	0.029 0.111 0.099	0.030 0.112 0.102	0.079 0.112 0.098	0.036 0.122 0.105	0.036 0.123 0.104	0.069 0.100 0.092	0.074 0.109 0.095	0.084 0.121 0.101
	25	0.6	0.6	0.043 0.228 0.093	0.005 0.069 0.094	0.005 0.072 0.096	0.004 0.066 0.094	0.004 0.067 0.095	0.040 0.101 0.091	0.006 0.080 0.111	0.004 0.080 0.106	0.033 0.079 0.075	0.038 0.100 0.84	0.044 0.117 0.093
		0.41	2	0.104 0.104 0.011	0.048 0.107 0.091	0.043 0.109 0.090	0.040 0.107 0.091	0.038 0.107 0.089	0.103 0.100 0.089	0.050 0.118 0.097	0.048 0.118 0.091	0.075 0.091 0.083	0.082 0.098 0.086	0.100 0.107 0.091
		0.31	3.75	0.095 0.100 0.010	0.077 0.104 0.091	0.072 0.103 0.089	0.069 0.105 0.091	0.065 0.105 0.088	0.089 0.100 0.089	0.080 0.110 0.094	0.078 0.110 0.087	0.082 0.094 0.086	0.085 0.098 0.087	0.091 0.104 0.091
	30	0.54	0.9	0.058 0.199 0.035	0.011 0.085 0.084	0.010 0.090 0.085	0.009 0.083 0.084	0.009 0.084 0.085	0.055 0.100 0.080	0.012 0.097 0.095	0.012 0.098 0.093	0.055 0.100 0.078	0.055 0.100 0.078	0.059 0.103 0.083
	40	0.69	0.35	0.024 0.182 0.145	0.002 0.027 0.074	0.002 0.028 0.079	0.001 0.023 0.074	0.002 0.023 0.078	0.015 0.080 0.069	0.002 0.031 0.096	0.002 0.031 0.095	0.013 0.057 0.055	0.015 0.082 0.067	0.016 0.097 0.073
		0.54	0.9	0.056 0.196 0.034	0.009 0.076 0.071	0.009 0.084 0.072	0.007 0.075 0.071	0.007 0.075 0.073	0.048 0.100 0.069	0.010 0.089 0.080	0.009 0.089 0.079	0.040 0.067 0.062	0.049 0.081 0.068	0.050 0.100 0.072
	45	0.69	0.35	0.016 0.185 0.137	0.001 0.025 0.069	0.001 0.028 0.074	0.001 0.022 0.069	0.001 0.022 0.073	0.0.14 0.100 0.100	0.001 0.029 0.089	0.001 0.029 0.088	0.012 0.054 0.052	0.015 0.078 0.063	0.016 0.092 0.069
		0.65	0.45	0.019 0.229 0.088	0.002 0.046 0.069	0.001 0.052 0.073	0.001 0.042 0.069	0.001 0.042 0.073	0.024 0.106 0.065	0.002 0.054 0.087	0.002 0.054 0.086	0.019 0.057 0.054	0.026 0.078 0.064	0.026 0.089 0.068
		0.6	0.6	0.035 0.233 0.058	0.004 0.056 0.068	0.004 0.064 0.070	0.002 0.053 0.068	0.003 0.053 0.070	0.033 0.076 0.065	0.004 0.066 0.080	0.004 0.066 0.079	0.027 0.059 0.057	0.035 0.077 0.064	0.035 0.087 0.068
		0.3	4	0.083 0.010 0.010	0.065 0.077 0.066	0.068 0.078 0.066	0.059 0.078 0.066	0.059 0.078 0.065	0.068 0.075 0.066	0.069 0.081 0.069	0.069 0.081 0.066	0.063 0.071 0.064	0.069 0.074 0.065	0.070 0.077 0.066
	50	0.65	0.45	0.023 0.227 0.093	0.001 0.041 0.065	0.001 0.047 0.069	0.001 0.037 0.065	0.001 0.037 0.068	0.021 0.100 0.062	0.002 0.048 0.081	0.001 0.048 0.080	0.017 0.054 0.051	0.022 0.100 0.060	0.022 0.084 0.065
		0.6	0.6	0.037 0.233 0.047	0.004 0.055 0.065	0.003 0.063 0.067	0.002 0.051 0.064	0.002 0.052 0.067	0.033 0.100 0.062	0.004 0.065 0.078	0.004 0.066 0.076	0.027 0.056 0.053	0.035 0.074 0.061	0.034 0.082 0.065
		0.48	1.3	0.080 0.096 0.010	0.018 0.078 0.063	0.018 0.086 0.064	0.013 0.079 0.063	0.013 0.079 0.064	0.058 0.072 0.062	0.019 0.089 0.069	0.018 0.089 0.068	0.049 0.062 0.057	0.063 0.072 0.061	0.061 0.077 0.063
		0.45	1.65	0.098 0.075 0.010	0.028 0.076 0.063	0.028 0.083 0.063	0.021 0.078 0.063	0.020 0.078 0.064	0.062 0.071 0.062	0.020 0.086 0.068	0.028 0.086 0.067	0.054 0.063 0.058	0.067 0.072 0.061	0.065 0.077 0.063
	100	0.89	0.03	0.000 0.018 0.157	0.000 0.001 0.006	0.000 0.001 0.007	0.000 0.000 0.006	0.000 0.000 0.005	0.001 0.053 0.043	0.000 0.001 0.008	0.000 0.001 0.008	0.001 0.022 0.022	0.001 0.053 0.041	0.001 0.076 0.049
		0.84	0.07	0.000 0.043 0.337	0.000 0.003 0.023	0.000 0.002 0.029	0.000 0.001 0.023	0.000 0.001 0.025	0.001 0.053 0.100	0.000 0.002 0.036	0.000 0.002 0.037	0.001 0.027 0.027	0.002 0.055 0.042	0.001 0.071 0.048
		0.68	0.35	0.009 0.180 0.057	0.001 0.017 0.046	0.000 0.021 0.051	0.000 0.014 0.046	0.000 0.014 0.049	0.009 0.100 0.044	0.000 0.020 0.061	0.000 0.020 0.061	0.008 0.037 0.035	0.011 0.054 0.043	0.009 0.061 0.046
		0.6	0.6	0.029 0.229 0.010	0.003 0.048 0.045	0.002 0.061 0.047	0.001 0.044 0.045	0.001 0.044 0.047	0.026 0.051 0.044	0.003 0.059 0.054	0.003 0.059 0.054	0.022 0.040 0.038	0.031 0.053 0.043	0.025 0.058 0.045
**Weibull Shape>1 = 1.5**														
	10	0.49	1.7	0.097 0.160 0.109	0.063 0.167 0.170	0.047 0.144 0.158	0.060 0.163 0.170	0.077 0.172 0.169	0.110 0.155 0.136	0.065 0.182 0.202	0.068 0.188 0.190	0.101 0.126 0.091	0.090 0.146 0.120	0.131 0.191 0.157
	15	0.56	1.5	0.096 0.159 0.103	0.057 0.149 0.139	0.044 0.136 0.135	0.050 0.143 0.139	0.059 0.147 0.141	0.099 0.129 0.112	0.059 0.166 0.173	0.059 0.169 0.167	0.089 0.100 0.073	0.087 0.125 0.102	0.120 0.159 0.129
	20	0.44	2	0.092 0.100 0.022	0.089 0.134 0.112	0.075 0.131 0.111	0.079 0.132 0.112	0.083 0.133 0.113	0.098 0.112 0.098	0.093 0.150 0.135	0.098 0.151 0.132	0.090 0.093 0.073	0.091 0.110 0.093	0.111 0.130 0.109
	35	0.7	1	0.068 0.210 0.078	0.018 0.097 0.095	0.015 0.098 0.100	0.014 0.088 0.095	0.014 0.088 0.099	0.067 0.103 0.073	0.019 0.111 0.132	0.018 0.112 0.129	0.057 0.059 0.041	0.067 0.088 0.068	0.083 0.110 0.083
	40	0.7	1	0.066 0.207 0.081	0.015 0.095 0.087	0.013 0.098 0.094	0.011 0.086 0.087	0.013 0.087 0.092	0.066 0.081 0.069	0.016 0.109 0.122	0.016 0.110 0.121	0.056 0.055 0.039	0.068 0.083 0.065	0.082 0.103 0.078
Gamma Shape=1	10	0.4	1.6	0.092 0.179 0.108	0.047 0.151 0.163	0.036 0.132 0.153	0.044 0.147 0.163	0.056 0.152 0.162	0.099 0.155 0.138	0.049 0.163 0.186	0.050 0.168 0.176	0.090 0.129 0.108	0.083 0.146 0.125	0.117 0.185 0.153
		0.56	1.3	0.080 0.203 0.149	0.031 0.133 0.163	0.023 0.115 0.151	0.029 0.129 0.163	0.039 0.137 0.163	0.087 0.205 0.137	0.032 0.145 0.189	0.030 0.150 0.178	0.078 0.123 0.101	0.070 0.145 0.122	0.106 0.191 0.154
		0.44	1.9	0.097 0.159 0.108	0.060 0.160 0.161	0.046 0.140 0.151	0.056 0.156 0.161	0.066 0.161 0.160	0.099 0.155 0.139	0.062 0.172 0.181	0.062 0.177 0.171	0.098 0.133 0.113	0.088 0.146 0.126	0.121 0.180 0.152
	15	0.56	1.3	0.083 0.186 0.106	0.032 0.129 0.131	0.025 0.120 0.128	0.028 0.124 0.130	0.033 0.127 0.133	0.086 0.129 0.112	0.033 0.144 0.157	0.032 0.146 0.152	0.076 0.103 0.088	0.076 0.124 0.106	0.101 0.154 0.123
	20	0.5	1.6	0.101 0.137 0.027	0.052 0.126 0.109	0.044 0.124 0.108	0.045 0.123 0.109	0.049 0.124 0.110	0.100 0.112 0.098	0.055 0.141 0.129	0.056 0.142 0.126	0.082 0.093 0.079	0.086 0.110 0.094	0.103 0.128 0.107
		0.3	3.1	0.070 0.026 0.014	0.099 0.122 0.102	0.088 0.120 0.100	0.092 0.123 0.102	0.093 0.123 0.105	0.098 0.111 0.099	0.102 0.132 0.113	0.107 0.132 0.111	0.093 0.102 0.088	0.091 0.109 0.096	0.105 0.121 0.104
	25	0.65	0.9	0.059 0.219 0.099	0.013 0.088 0.103	0.011 0.088 0.107	0.010 0.082 0.103	0.013 0.083 0.106	0.059 0.101 0.087	0.014 0.101 0.134	0.015 0.102 0.130	0.051 0.074 0.061	0.058 0.101 0.081	0.072 0.124 0.096
		0.56	1.3	0.093 0.145 0.068	0.039 0.119 0.098	0.033 0.120 0.100	0.031 0.114 0.098	0.033 0.115 0.101	0.090 0.100 0.087	0.041 0.136 0.122	0.041 0.137 0.119	0.072 0.080 0.066	0.079 0.101 0.083	0.094 0.119 0.095
	35	0.66	0.9	0.063 0.219 0.068	0.015 0.081 0.087	0.012 0.084 0.092	0.011 0.073 0.087	0.011 0.074 0.090	0.060 0.106 0.074	0.015 0.093 0.116	0.015 0.094 0.113	0.048 0.062 0.051	0.058 0.087 0.071	0.067 0.105 0.081
	40	0.66	0.9	0.055 0.217 0.062	0.011 0.081 0.081	0.009 0.087 0.086	0.007 0.074 0.081	0.008 0.074 0.084	0.055 0.106 0.069	0.011 0.094 0.108	0.010 0.095 0.106	0.046 0.059 0.048	0.056 0.102 0.066	0.064 0.098 0.075
	45	0.66	0.9	0.052 0.195 0.067	0.010 0.080 0.075	0.009 0.087 0.080	0.007 0.0072 0.075	0.007 0.073 0.078	0.055 0.100 0.065	0.010 0.093 0.100	0.010 0.094 0.099	0.046 0.055 0.046	0.058 0.101 0.063	0.063 0.102 0.071
		0.39	2.3	0.074 0.011 0.010	0.076 0.084 0.069	0.077 0.088 0.070	0.066 0.085 0.069	0.066 0.085 0.070	0.070 0.075 0.064	0.080 0.094 0.080	0.081 0.094 0.079	0.063 0.066 0.055	0.071 0.075 0.064	0.076 0.083 0.068
	50	0.79	0.5	0.022 0.162 0.268	0.002 0.028 0.072	0.001 0.027 0.081	0.001 0.021 0.072	0.002 0.021 0.077	0.021 0.073 0.061	0.002 0.031 0.105	0.002 0.031 0.105	0.017 0.044 0.036	0.021 0.075 0.058	0.024 0.096 0.069
**Gamma Shape<1 = 0.5**														
	10	0.42	0.8	0.097 0.169 0.107	0.052 0.149 0.151	0.040 0.134 0.142	0.048 0.147 0.151	0.050 0.149 0.153	0.098 0.156 0.141	0.053 0.158 0.163	0.049 0.162 0.157	0.090 0.139 0.127	0.082 0.145 0.129	0.108 0.174 0.149
		0.4	0.9	0.100 0.161 0.100	0.058 0.152 0.151	0.045 0.137 0.142	0.055 0.149 0.151	0.055 0.152 0.153	0.101 0.156 0.142	0.059 0.159 0.161	0.056 0.163 0.156	0.093 0.141 0.129	0.084 0.145 0.129	0.107 0.172 0.148
	15	0.55	0.45	0.073 0.209 0.109	0.022 0.106 0.122	0.018 0.100 0.119	0.019 0.102 0.122	0.022 0.105 0.124	0.067 0.129 0.112	0.023 0.117 0.138	0.022 0.119 0.134	0.058 0.106 0.099	0.060 0.123 0.106	0.076 0.149 0.119
		0.48	0.6	0.089 0.169 0.104	0.037 0.126 0.119	0.029 0.121 0.116	0.032 0.123 0.119	0.034 0.125 0.123	0.081 0.129 0.112	0.038 0.138 0.131	0.037 0.140 0.129	0.072 0.111 0.102	0.072 0.124 0.107	0.089 0.145 0.118
		0.4	0.9	0.104 0.109 0.100	0.059 0.137 0.118	0.049 0.131 0.115	0.053 0.135 0.118	0.054 0.136 0.121	0.101 0.129 0.113	0.061 0.148 0.127	0.058 0.149 0.124	0.091 0.116 0.105	0.089 0.123 0.107	0.108 0.140 0.117
	20	0.53	0.45	0.079 0.194 0.085	0.024 0.105 0.103	0.019 0.104 0.102	0.019 0.102 0.103	0.019 0.102 0.105	0.070 0.112 0.098	0.025 0.118 0.117	0.024 0.119 0.114	0.058 0.093 0.086	0.063 0.110 0.094	0.074 0.127 0.104
		0.71	0.15	0.025 0.184 0.280	0.002 0.041 0.097	0.002 0.039 0.098	0.002 0.037 0.097	0.003 0.038 0.103	0.025 0.113 0.108	0.002 0.047 0.121	0.002 0.048 0.122	0.021 0.076 0.073	0.023 0.109 0.089	0.029 0.143 0.108
		0.47	0.6	0.097 0.153 0.037	0.041 0.116 0.101	0.035 0.115 0.100	0.035 0.113 0.101	0.033 0.114 0.104	0.081 0.112 0.099	0.043 0.128 0.112	0.039 0.129 0.110	0.071 0.097 0.089	0.075 0.109 0.095	0.087 0.124 0.103
	25	0.63	0.25	0.052 0.222 0.119	0.009 0.068 0.097	0.008 0.068 0.100	0.007 0.062 0.097	0.007 0.063 0.100	0.041 0.101 0.101	0.010 0.078 0.120	0.008 0.079 0.116	0.035 0.077 0.070	0.039 0.101 0.100	0.045 0.120 0.103
		0.58	0.35	0.064 0.214 0.087	0.014 0.086 0.094	0.012 0.089 0.095	0.011 0.082 0.094	0.012 0.083 0.095	0.054 0.101 0.088	0.015 0.098 0.110	0.014 0.099 0.105	0.047 0.081 0.074	0.053 0.100 0.084	0.060 0.116 0.093
	35	0.64	0.25	0.037 0.227 0.114	0.004 0.059 0.080	0.004 0.063 0.084	0.003 0.054 0.080	0.004 0.054 0.084	0.034 0.106 0.074	0.005 0.069 0.101	0.005 0.069 0.099	0.028 0.064 0.060	0.034 0.087 0.071	0.037 0.102 0.079
		0.59	0.35	0.056 0.218 0.066	0.011 0.076 0.078	0.009 0.082 0.081	0.007 0.072 0.078	0.008 0.073 0.081	0.048 0.086 0.074	0.011 0.088 0.094	0.010 0.089 0.092	0.040 0.068 0.063	0.048 0.087 0.072	0.052 0.098 0.078
	45	0.8	0.07	0.000 0.078 0.355	0.00 0.008 0.053	0.000 0.007 0.060	0.000 0.005 0.053	0.000 0.005 0.056	0.005 0.077 0.065	0.000 0.009 0.076	0.000 0.009 0.076	0.005 0.043 0.043	0.005 0.077 0.060	0.005 0.103 0.072
	50	0.8	0.07	0.000 0.079 0.354	0.000 0.006 0.052	0.000 0.006 0.059	0.000 0.004 0.052	0.000 0.005 0.055	0.005 0.073 0.061	0.000 0.007 0.075	0.000 0.006 0.075	0.005 0.041 0.039	0.005 0.074 0.057	0.006 0.097 0.068
	100	0.92	0.01	0.000 0.029 0.091	0.000 0.001 0.004	0.000 0.001 0.004	0.000 0.001 0.004	0.000 0.001 0.003	0.001 0.053 0.043	0.000 0.001 0.005	0.000 0.001 0.005	0.001 0.018 0.019	0.001 0.052 0.039	0.001 0.079 0.049
Gamma	10	0.51	2.5	0.104 0.176 0.121	0.050 0.157 0.167	0.037 0.135 0.155	0.047 0.152 0.167	0.058 0.158 0.164	0.105 0.155 0.136	0.052 0.170 0.197	0.050 0.175 0.183	0.095 0.126 0.094	0.087 0.145 0.121	0.127 0.189 0.156
shape>1 = 1.5	15	0.46	3	0.101 0.112 0.101	0.071 0.149 0.132	0.057 0.139 0.128	0.064 0.146 0.132	0.068 0.148 0.134	0.108 0.129 0.112	0.074 0.164 0.157	0.074 0.167 0.152	0.099 0.108 0.085	0.096 0.125 0.106	0.124 0.150 0.124
		0.59	2	0.089 0.189 0.122	0.038 0.132 0.137	0.030 0.120 0.134	0.034 0.126 0.137	0.040 0.130 0.139	0.088 0.129 0.112	0.040 0.147 0.170	0.039 0.150 0.165	0.078 0.099 0.075	0.077 0.125 0.103	0.107 0.160 0.128
	20	0.59	2	0.090 0.177 0.060	0.037 0.126 0.120	0.030 0.121 0.120	0.032 0.120 0.120	0.034 0.122 0.122	0.091 0.112 0.098	0.039 0.142 0.151	0.037 0.144 0.148	0.076 0.086 0.066	0.079 0.111 0.091	0.102 0.138 0.111
		0.69	1.5	0.062 0.193 0.160	0.017 0.100 0.125	0.013 0.093 0.125	0.014 0.092 0.125	0.020 0.094 0.126	0.069 0.113 0.107	0.018 0.113 0.162	0.019 0.114 0.158	0.059 0.079 0.056	0.063 0.112 0.086	0.087 0.146 0.112
	25	0.6	2	0.091 0.158 0.040	0.039 0.122 0.104	0.032 0.121 0.106	0.031 0.116 0.104	0.032 0.117 0.107	0.093 0.101 0.086	0.040 0.139 0.135	0.039 0.140 0.132	0.073 0.077 0.058	0.080 0.101 0.081	0.099 0.123 0.097
		0.68	1.5	0.068 0.191 0.107	0.018 0.103 0.111	0.015 0.100 0.114	0.015 0.095 0.111	0.017 0.096 0.113	0.068 0.104 0.100	0.019 0.117 0.147	0.019 0.118 0.144	0.059 0.071 0.052	0.066 0.102 0.080	0.086 0.128 0.101
	30	0.47	3	0.099 0.042 0.013	0.075 0.111 0.088	0.068 0.114 0.089	0.063 0.110 0.088	0.064 0.111 0.090	0.090 0.092 0.073	0.078 0.126 0.108	0.079 0.127 0.106	0.077 0.076 0.060	0.084 0.092 0.078	0.095 0.106 0.086
		0.6	2	0.089 0.145 0.019	0.032 0.115 0.094	0.028 0.118 0.097	0.026 0.110 0.094	0.026 0.111 0.097	0.081 0.100 0.079	0.034 0.133 0.123	0.032 0.133 0.121	0.069 0.070 0.053	0.079 0.093 0.076	0.095 0.112 0.088
	35	0.35	4	0.053 0.012 0.010	0.088 0.095 0.079	0.085 0.098 0.080	0.080 0.096 0.079	0.080 0.096 0.081	0.077 0.085 0.074	0.093 0.106 0.094	0.096 0.106 0.092	0.071 0.074 0.060	0.076 0.085 0.073	0.085 0.094 0.079
		0.59	2	0.095 0.141 0.022	0.036 0.109 0.086	0.032 0.113 0.090	0.029 0.103 0.087	0.028 0.104 0.089	0.080 0.106 0.074	0.038 0.126 0.114	0.036 0.126 0.112	0.068 0.065 0.050	0.080 0.087 0.070	0.091 0.103 0.082
	40	0.21	7	0.014 0.010 0.012	0.076 0.084 0.072	0.074 0.084 0.071	0.074 0.085 0.072	0.074 0.085 0.073	0.069 0.079 0.069	0.079 0.090 0.079	0.080 0.090 0.078	0.067 0.073 0.062	0.070 0.079 0.069	0.074 0.084 0.072
		0.37	4	0.052 0.012 0.010	0.084 0.089 0.074	0.082 0.092 0.075	0.075 0.091 0.074	0.076 0.091 0.076	0.072 0.079 0.069	0.088 0.100 0.088	0.090 0.100 0.087	0.067 0.069 0.056	0.073 0.079 0.069	0.080 0.088 0.074
		0.46	3	0.087 0.019 0.011	0.079 0.095 0.076	0.076 0.100 0.077	0.067 0.096 0.076	0.066 0.096 0.078	0.075 0.079 0.069	0.083 0.109 0.093	0.084 0.109 0.092	0.067 0.066 0.052	0.076 0.081 0.068	0.084 0.091 0.075
	45	0.14	11	0.010 0.012 0.010	0.068 0.077 0.067	0.069 0.077 0.067	0.067 0.078 0.067	0.067 0.078 0.067	0.065 0.075 0.065	0.070 0.081 0.071	0.071 0.081 0.070	0.063 0.071 0.061	0.063 0.074 0.065	0.067 0.078 0.067
		0.59	2	0.096 0.132 0.011	0.035 0.096 0.075	0.032 0.104 0.078	0.026 0.093 0.075	0.026 0.093 0.078	0.070 0.106 0.065	0.037 0.113 0.099	0.036 0.113 0.098	0.060 0.058 0.043	0.070 0.099 0.063	0.081 0.091 0.072
	50	0.35	4	0.035 0.010 0.010	0.077 0.079 0.065	0.078 0.082 0.067	0.070 0.081 0.066	0.070 0.081 0.067	0.065 0.071 0.062	0.081 0.089 0.077	0.083 0.089 0.077	0.060 0.062 0.050	0.066 0.072 0.062	0.071 0.079 0.065
		0.59	2	0.099 0.110 0.010	0.037 0.093 0.071	0.034 0.102 0.074	0.027 0.091 0.071	0.027 0.091 0.073	0.070 0.100 0.062	0.039 0.110 0.094	0.037 0.110 0.092	0.059 0.055 0.042	0.074 0.099 0.061	0.080 0.086 0.068

### Results of Simulation

We used the survival times {y_i_} and indicator variables {σ_i_} and computed the Kaplan–Meier survival function, the Greenwood, Peto, regular, adjusted hybrid, weighted variance, and different combinations of weighted Kaplan–Meier functions. Also the simulated *SD* is calculated by the Borkowf method. The average of all ten *SEs* are compared with the values of simulated *SD* at three quartiles namely Q_1_, Q_2_, Q_3_. With different censoring percentages, average of the Greenwood's, Peto's, regular, and hybrid variances are smaller substantially in the first quartile, while the average weighted *SEs* are approximately equal to the stimulated *SDs* (target values). The combination of the Kaplan–Meier survival probability with variance of weighted Kaplan–Meier did not give as close results as obtained by the weighted Kaplan–Meier. Similar, results obtained from the combination of shrunken survival probabilities with the weighted variance factor. The efficiency of the Greenwood *SE* for a very small sample sizes is achieved by taking the weighted probabilities in the formula. Similarly, by taking the weighted probability, we got the better results of the Peto too. Improvement also occurred in the adjusted hybrid method by taking the weighted survival probabilities.

Also in the second and third quartiles, the results of the weighted method matches closer to the target values as compared to the Greenwood, Peto, regular, and hybrid methods, while in some cases, these four methods substantially over estimate the true values of these target values.

The combination of the weighted survival probabilities and variance factors gave the better results. Also for comparatively large sample sizes 45, 50, and 100, weighted variance gave the much better result. So it is and in some situations (moderate and heavy censoring), its combination with the hybrid and Peto's are the best methods.

## Analysis of Datasets

We used different datasets from real life, in order to make it more practical and easy. The details of the datasets are described below:

### Application on Thalassaemia Data

The data of 70 patients were collected from the Fatimid Foundation, Peshawar (Pakistan). Fatimid foundation, a non-profit charity organization, is the pioneer in voluntary blood transfusion services in Pakistan. Thalassaemia is an inherited disorder with an abnormality in one or more of the globin genes. The time is taken in months of the treatment.

Out of 70 patients, 56 were censored and 14 events occurred. This means there is heavy censoring in the data, which is also due to the fact that the treatment of the disease is a life-long. For this reason, most people stop the treatment. The data are given below indicating months of treatment.

3+, 3+, 5+, 5+, 15+, 15+, 16+, 27, 30+, 30+, 46+, 50+, 50+, 50+, 53+, 56+, 56+, 56+, 58+, 60+, 60+, 61+, 64, 64, 70+, 70+, 72+, 72+, 72+, 77+, 79+, 80+, 80+, 80+, 94+, 94+, 94+, 96+, 96+, 102, 102, 108+, 108+, 108+, 111, 111, 113+, 114+, 114+, 117+, 117+, 117+, 122, 122, 126+, 127+, 129, 138+, 138+, 142+, 145+, 155+, 156, 161+, 164+, 165, 165, 173, 173+, 175.

Where a plus sign denotes a censored observation. The results of the analysis are given in (Table 2).

**Table 2:** Estimated survival functions and *SE*s using the five variance estimation methods and their combinations.

**s.no**.	**Time**	**r_K_**	**d**	**c**	**mx**	**N-mx**	**S_KM_**	**S_W_**	S_KM*_	**SE_G_**	**SE_P_**	**SE_H_**	SE_H_*	**SE_W_**	**SE_KMW_**	SE_KM*W_	**SE_WG_**	**SE_WP_**	**SE_WH_**
1	3	70	0	2	2	68	1.0000	0.9916	0.9929	0.0000	0.0000	0.0000	0.0102	0.0108	0.0109	0.0108	0.0000	0.0108	0.0110
2	5	68	0	2	4	66		0.9833				0.0000	0.0103	0.0153	0.0155	0.0154	0.0000	0.0154	0.0158
3	15	66	0	2	6	64		0.9750				0.0000	0.0105	0.0187	0.0192	0.0190	0.0000	0.0190	0.0195
4	16	64	0	1	7	63		0.9667				0.0000	0.0106	0.0216	0.0223	0.0222	0.0000	0.0220	0.0226
5	27	63	1	0	7	63	0.9841	0.9514	0.9772	0.0157	0.0156	0.0157	0.0188	0.0261	0.0270	0.0269	0.0152	0.0264	0.0271
6	30	62	0	2	9	61		0.9435				0.0160	0.0191	0.0281	0.0293	0.0291	0.0151	0.0285	0.0296
7	46	60	0	1	10	60		0.9356				0.0161	0.0193	0.0300	0.0315	0.0313	0.0150	0.0306	0.0317
8	50	59	0	3	13	57		0.9279				0.0166	0.0198	0.0317	0.0336	0.0333	0.0148	0.0324	0.0343
9	53	56	0	1	14	56		0.9201				0.0167	0.0199	0.0333	0.0357	0.0354	0.0147	0.0348	0.0362
10	56	55	0	3	17	53		0.9125				0.0172	0.0205	0.0349	0.0376	0.0374	0.0146	0.0364	0.0388
11	58	52	0	1	18	52		0.9047				0.0173	0.0207	0.0365	0.0397	0.0394	0.0145	0.0387	0.0407
12	60	51	0	2	20	50		0.8971				0.0177	0.0211	0.0379	0.0416	0.0413	0.0144	0.0403	0.0430
13	61	49	0	1	21	49		0.8895				0.0179	0.0213	0.0394	0.0436	0.0433	0.0142	0.0422	0.0448
14	64	48	2	0	21	49	0.9431	0.8524	0.9368	0.0321	0.0325	0.0331	0.0348	0.0456	0.0505	0.0501	0.0291	0.0473	0.0507
15	70	46	0	2	23	47		0.8453				0.0338	0.0355	0.0467	0.0521	0.0517	0.0288	0.0490	0.0527
16	72	44	0	3	26	44		0.8382				0.0349	0.0367	0.0477	0.0537	0.0533	0.0286	0.0508	0.0555
17	77	41	0	1	27	43		0.8309				0.0353	0.0371	0.0488	0.0554	0.0550	0.0283	0.0534	0.0572
18	79	40	0	1	28	42		0.8237				0.0357	0.0375	0.0498	0.0571	0.0567	0.0281	0.0547	0.0588
19	80	39	0	3	31	39		0.8168				0.0371	0.0390	0.0508	0.0587	0.0583	0.0278	0.0560	0.0619
20	94	36	0	3	34	36		0.8097				0.0386	0.0406	0.0519	0.0605	0.0601	0.0276	0.0589	0.0654
21	96	33	0	2	36	34		0.8023				0.0397	0.0417	0.0531	0.0625	0.0621	0.0273	0.0621	0.0683
22	102	31	2	0	36	34	0.8823	0.7505	0.8768	0.0513	0.0544	0.0553	0.0564	0.0610	0.0717	0.0713	0.0437	0.0673	0.0742
23	108	29	0	3	39	31		0.7435				0.0579	0.0590	0.0619	0.0735	0.0730	0.0433	0.0699	0.0784
24	111	26	2	0	39	31	0.8144	0.6863	0.8099	0.0661	0.0688	0.0698	0.0705	0.0691	0.0820	0.0815	0.0557	0.0754	0.0833
25	113	24	0	1	40	30		0.6794				0.0710	0.0716	0.0698	0.0837	0.0832	0.0552	0.0785	0.0852
26	114	23	0	2	42	28		0.6726				0.0735	0.0742	0.0705	0.0853	0.0849	0.0546	0.0802	0.0887
27	117	21	0	3	45	25		0.6658				0.0778	0.0785	0.0713	0.0872	0.0867	0.0541	0.0840	0.0943
28	122	18	2	0	45	25	0.7239	0.5918	0.7207	0.0842	0.0897	0.0894	0.0897	0.0803	0.0982	0.0978	0.0689	0.0891	0.0983
29	126	16	0	1	46	24		0.5849				0.0913	0.0916	0.0809	0.1001	0.0997	0.0681	0.0942	0.1006
30	127	15	0	1	47	23		0.5782				0.0932	0.0935	0.0815	0.1021	0.1016	0.0673	0.0970	0.1030
31	129	14	1	0	47	23	0.6722	0.5369	0.6697	0.0927	0.1029	0.0979	0.0981	0.0855	0.1071	0.1067	0.0741	0.0977	0.1040
32	138	13	0	2	49	21		0.5310				0.1024	0.1026	0.0860	0.1088	0.1084	0.0733	0.1009	0.1089
33	142	11	0	1	50	20		0.5248				0.1050	0.1052	0.0866	0.1110	0.1106	0.0724	0.1091	0.1117
34	145	10	0	1	51	19		0.5188				0.1077	0.1079	0.0874	0.1133	0.1128	0.0716	0.1138	0.1146
35	155	9	0	1	52	18		0.5131				0.1106	0.1109	0.0883	0.1157	0.1152	0.0708	0.1193	0.1178
36	156	8	1	0	52	18	0.5882	0.4489	0.5869	0.1130	0.1335	0.1160	0.1161	0.0978	0.1281	0.1279	0.0862	0.1178	0.1172
37	161	7	0	1	53	17		0.4444				0.1194	0.1194	0.0983	0.1301	0.1298	0.0853	0.1252	0.1205
38	164	6	0	1	54	16		0.4401				0.1230	0.1231	0.0989	0.1322	0.1319	0.0845	0.1344	0.1241
39	165	5	2	0	54	16	0.3529	0.2641	0.3550	0.1456	0.1270	0.1195	0.1196	0.1132	0.1513	0.1522	0.1090	0.1013	0.1102
40	173	3	1	1	55	15	0.2353	0.1744	0.2391	0.1366	0.1188	0.1095	0.1101	0.1035	0.1396	0.1418	0.1012	0.0915	0.0980
41	175	1	0	1	56	14		0.1719				0.1134	0.1140	0.1040	0.1424	0.1446	0.0998	0.1564	0.1008

The first column gives the serial number which we used in the computation of the weighted formula, the second column is the time in months, and the third column is the number of persons at risk at different times. The following two columns denote the events and censoring at each time. mx denotes the cumulative sum of censoring; the next column is the subtraction of cumulative censoring from the total number of patients. The following three columns denote the Kaplan–Meier survival probabilities, survival probabilities based on weighted Kaplan–Meier, and survival probabilities based on shrunken Kaplan–Meier. After these three columns, the following ten columns show the ten different combinations of *SEs*.

If we look at the comparison of the new method with the others, then we see that our method gives the estimates as well as the *SE* at each point whether the event occurred or not. Its range is the same as that of Kaplan–Meier survival function, i.e., 0 and 1. Thus, unlike the shrunken survival function it starts from 1 for *t* ≤ *t*_(1)_. If we compare it with the others, we see that from the start its *SE* is greater than the *SE* of adjusted hybrid *SE*, which is also due to the fact that it includes the probabilities of all points which affect it. On the other hand, the patterns of regular and adjusted hybrid *SEs* are the same as described by Borkowf (5), since the Greenwood's and Peto's variance estimators can substantially under estimate the true variance in the left and right tails of the survival distribution, in case of moderate or heavy censoring. In the extreme lower part, the *SEs* of regular and adjusted hybrid just like the weighted *SEs* are smaller than the Greenwood's and Peto's *SEs*, so they also underestimate the variance on the right tail. Therefore, we need to find such a method which yields the better results compared to these methods. Thus we take the ${\overset{\hat{\;}}{S}}_{KM}$ probabilities instead of ${\overset{\hat{\;}}{S}}_{W}$showing immediate effects on the results and giving much better results on both tails than the other methods discussed above.

For more substantial conclusions, we use ${\overset{\hat{\;}}{S}}_{KM^{*}}$ instead of ${\overset{\hat{\;}}{S}}_{W}$ in our formula, which also gives the same pattern as by taking ${\overset{\hat{\;}}{S}}_{KM}$ instead of${\overset{\hat{\;}}{S}}_{W}$. This enables another choice of using it.

In order to determine the effects of S_W_ on the variances of Greenwood's, Peto's and regular variance, we replace S_KM_ by S_w_. Replacing it in the Greenwood's formula does not give a satisfactory result, as it actually underestimates the values at each point. This is due to the fact that ${\overset{\hat{\;}}{S}}_{W}\leq {\overset{\hat{\;}}{S}}_{KM}$. The case of Peto's variance is different from Greenwood's, yielding better results in the start but being smaller in the end than the Peto's based on S_KM_. It is the same case is repeated with the regular variance when using SW in the formula, i.e., it is smaller in the end. If we take the $\overset{\wedge }{S_{KM}}$ and $\overset{\wedge }{S_{KM*}}$, we get the best result.

### Case of Events Only in Leukemia Dataset

To further elaborate more these methods, the second group of the same leukemia data is considered, which consists of events only. The leukemia data are taken from Freireich et al. (8). The dataset is given below:

1, 1, 2, 2, 3, 4, 4, 5, 5, 8, 8, 8, 8, 11, 11, 12, 12, 15, 17, 22, 23 The analysis is shown in (Table 3).

**Table 3:** Estimated survival functions and *SE*s using the ten different methods from leukemia data.

**s.no**.	**Time**	**r_**k**_**	**d**	**c**	**mx**	**n-mx**	**S_**KM**_**	S_KM*_	**S_**W**_**	**SE_**G**_**	**SE_**P**_**	**SE_**H**_**	${\text{SE}}_{\text{H}}^{*}$	**SE_**W**_**	**SE_**KMW**_**	SE_KM*W_	**SE_**WG**_**	**SE_**WP**_**	**SE_**WH**_**
1	1	21	2	0	0	21	0.9048	0.8855	0.9048	0.0641	0.0609	0.0641	0.0695	0.0641	0.0641	0.0627	0.0641	0.0609	0.0641
2	2	19	2	0	0	21	0.8095	0.7948	0.8095	0.0857	0.0811	0.0857	0.0881	0.0857	0.0857	0.0841	0.0857	0.0811	0.0857
3	3	17	1	0	0	21	0.7619	0.7494	0.7619	0.0929	0.0902	0.0929	0.0946	0.0929	0.0929	0.0914	0.0929	0.0902	0.0929
4	4	16	2	0	0	21	0.6667	0.6587	0.6667	0.1029	0.0962	0.1029	0.1035	0.1029	0.1029	0.1016	0.1029	0.0962	0.1029
5	5	14	2	0	0	21	0.5714	0.5680	0.5714	0.1080	0.1000	0.1080	0.1081	0.1080	0.1080	0.1073	0.1080	0.1000	0.1080
6	8	12	4	0	0	21	0.3810	0.3866	0.3810	0.1060	0.0865	0.1060	0.1063	0.1060	0.1060	0.1075	0.1060	0.0865	0.1060
7	11	8	2	0	0	21	0.2857	0.2959	0.2857	0.0986	0.0854	0.0986	0.0996	0.0986	0.0986	0.1021	0.0986	0.0854	0.0986
8	12	6	2	0	0	21	0.1905	0.2052	0.1905	0.0857	0.0700	0.0857	0.0881	0.0857	0.0857	0.0923	0.0857	0.0700	0.0857
9	15	4	1	0	0	21	0.1429	0.1599	0.1429	0.0764	0.0661	0.0764	0.0800	0.0764	0.0764	0.0855	0.0764	0.0661	0.0764
10	17	3	1	0	0	21	0.0952	0.1145	0.0952	0.0641	0.0523	0.0641	0.0695	0.0641	0.0641	0.0770	0.0641	0.0523	0.0641
11	22	2	1	0	0	21	0.0476	0.0692	0.0476	0.0465	0.0329	0.0465	0.0554	0.0465	0.0465	0.0675	0.0465	0.0329	0.0465
12	23	1	1	0	0	21	0.0000	0.0238	0.0000	0.0000	0.0000	0.0000	0.0333	0.0000	0.0000	0.0232	0.0000	0.0000	0.0000

In case of events only, S_KM_ = S_W_, while the shrunken Kaplan–Meier survival function ($\overset{\wedge }{S_{KM*}}$) is different from these. Similarly, SE_G_= SE_H_ = SE_W_ = SE_KMW_ = SE_WG_ = SE_WH_ and variance reduced to binomial variance. Also SE_P_ = SE_WP_, because in case of no censoring time, S_KM_ = S_W_, while SE_H*_ and SE_KM*W_ differ. SE_H*_ is greater in all situations from SE_G_ and SE_P_. While the comparison of SE_H*_ and SE_KM*W_ gives a mixed picture, in the start, the former is larger but in the later stages SE_KM*W_ is larger. While in case of events only, binomial variance is considered to be the best choice, so it overestimates the variance and Peto's method under estimate.

### Case of Censored Data Only

In order to check the performance of these methods in case of censored data only, we interchange the role of censoring and occurrence of events in the above example to get the Table 4.

**Table 4:** Estimated survival functions and *SE*s of the leukemia data containing only censoring time.

**s.no**.	**time**	**Rk**	**e**	**c**	**mx**	**n-mx**	**S_KM_**	S_KM*_	**S_W_**	**SE_G_**	**SE_P_**	**SE_H_**	SE_H_*	**SE_W_**	**SE_KMW_**	SE_KM*W_	**SE_WG_**	**SE_WP_**	**SE_WH_**
0	21	0	0	0	0	21	1.0000	0.9760	1.0000	0.0000	0.0000	0.0000	0.0330	0.0000	0.0000	0.0000	0.0000	0.0000	0.0000
1	21	1	0	2	2	19			0.9728				0.0351	0.0345	0.0355	0.0347		0.0350	0.0373
2	19	2	0	2	4	17			0.9460				0.0371	0.0489	0.0517	0.0504		0.0504	0.0548
3	17	3	0	1	5	16			0.9195				0.0383	0.0601	0.0654	0.0638		0.0633	0.0680
4	16	4	0	2	7	14			0.8948				0.0409	0.0687	0.0768	0.0750		0.0725	0.0820
5	14	5	0	2	9	12			0.8705				0.0442	0.0768	0.0883	0.0862		0.0837	0.0969
6	12	8	0	4	13	8			0.8463				0.0541	0.0848	0.1002	0.0978		0.0958	0.1275
7	8	11	0	2	15	6			0.8161				0.0625	0.0978	0.1198	0.1169		0.1237	0.1582
8	6	12	0	2	17	4			0.7837				0.0765	0.1128	0.1439	0.1404		0.1488	0.2059
9	4	15	0	1	18	3			0.7464				0.0884	0.1336	0.1790	0.1747		0.1879	0.2512
10	3	17	0	1	19	2			0.7108				0.1082	0.1544	0.2172	0.2119		0.2207	0.3206
11	2	22	0	1	20	1			0.6770				0.1530	0.1789	0.2643	0.2579		0.2721	0.4676
12	1	23	0	1	21	0			0.6448				0.0000	0.2188	0.3394	0.3312		0.3843	0.0000

As traditionally, the probabilities of Kaplan–Meier and shrunken are struck to one and 0.976, since *n* = 21, i.e., these are the probabilities at time *t* ≤ *t*_(1)_. While the new method gives the estimate at each point affecting the *SE* too. SE_W_ gives a smaller value at the start but then increases toward the end. The same is the case with the combination of this formula with the S_KM_ and S_KM*_. *SE* of regular hybrid variance using the S_W_ gives much bigger values than the all others, while SE_G_ gives a value of zero for each case.

### Cancer Dataset From 1,207 Patients With 94% Censoring

Here, we consider a bigger dataset containing 1,207 patients with very heavy censoring (94%) obtained from the SPSS (9) data. Some of the information regarding the analysis of this data are given in (Table 5).

**Table 5:** Selected estimated survival functions and *SEs* of the cancer data.

**s.no**.	**Time**	**r_**k**_**	**e**	**c**	**mx**	**n-mx**	**S_KM_**	S_KM*_	**S_W_**	**SE_G_**	**SE_P_**	**SE_H_**	${\text{SE}}_{\text{H}}^{*}$	**SE_W_**	**SE_KMW_**	SE_KM*W_	**SE_WG_**	**SE_WP_**	**SE_WH_**
1	2.63	1207	1	0	0	1207	0.9992	0.9988	0.9992	0.0008	0.0008	0.0008	0.0008	0.0010	0.0008	0.0008	0.0008	0.0008	0.0008
2	3.00	1206	0	2	2	1205	0.9992	0.9988	0.9985	0.0008	0.0008	0.0011	0.0008	0.0010	0.0011	0.0011	0.0008	0.0011	0.0011
3	3.27	1204	0	1	3	1204	0.9992	0.9988	0.9978	0.0008	0.0008	0.0013	0.0008	0.0010	0.0013	0.0013	0.0008	0.0013	0.0013
4	3.37	1203	0	1	4	1203	0.9992	0.9988	0.9972	0.0008	0.0008	0.0015	0.0008	0.0010	0.0015	0.0015	0.0008	0.0015	0.0015
5	3.53	1202	0	1	5	1202	0.9992	0.9988	0.9965	0.0008	0.0008	0.0017	0.0008	0.0010	0.0017	0.0017	0.0008	0.0017	0.0017
146	15.77	1012	0	1	187	1020	0.9917	0.9913	0.9025	0.0028	0.0028	0.0087	0.0028	0.0029	0.0096	0.0096	0.0025	0.0089	0.0093
147	15.80	1011	0	1	188	1019	0.9917	0.9913	0.9019	0.0028	0.0028	0.0087	0.0028	0.0029	0.0096	0.0096	0.0025	0.0089	0.0093
148	15.83	1010	0	1	189	1018	0.9917	0.9913	0.9013	0.0028	0.0028	0.0087	0.0028	0.0029	0.0096	0.0096	0.0025	0.0089	0.0093
149	15.90	1009	0	1	190	1017	0.9917	0.9913	0.9007	0.0028	0.0028	0.0088	0.0029	0.0029	0.0097	0.0097	0.0025	0.0089	0.0094
150	16.00	1008	0	1	191	1016	0.9917	0.9913	0.9001	0.0028	0.0028	0.0088	0.0029	0.0029	0.0097	0.0097	0.0025	0.0090	0.0094
275	25.97	843	0	1	343	864	0.9777	0.9773	0.8189	0.0047	0.0050	0.0115	0.0050	0.0051	0.0138	0.0138	0.0040	0.0120	0.0131
276	26.00	842	0	2	345	862	0.9777	0.9773	0.8184	0.0047	0.0050	0.0115	0.0050	0.0051	0.0138	0.0138	0.0040	0.0120	0.0131
277	26.13	840	1	0	345	862	0.9765	0.9761	0.8174	0.0049	0.0052	0.0116	0.0052	0.0052	0.0138	0.0138	0.0041	0.0121	0.0132
278	26.17	839	0	2	347	860	0.9765	0.9761	0.8168	0.0049	0.0052	0.0116	0.0052	0.0052	0.0138	0.0138	0.0041	0.0121	0.0132
279	26.30	837	0	1	348	859	0.9765	0.9761	0.8163	0.0049	0.0052	0.0116	0.0052	0.0052	0.0139	0.0139	0.0041	0.0121	0.0132
395	37.43	693	0	1	482	725	0.9640	0.9636	0.7490	0.0062	0.0069	0.0133	0.0069	0.0070	0.0171	0.0171	0.0048	0.0143	0.0161
396	37.47	692	0	1	483	724	0.9640	0.9636	0.7485	0.0062	0.0069	0.0133	0.0069	0.0070	0.0171	0.0171	0.0048	0.0143	0.0161
397	37.50	691	0	1	484	723	0.9640	0.9636	0.7479	0.0062	0.0069	0.0133	0.0069	0.0070	0.0172	0.0171	0.0048	0.0143	0.0161
398	37.57	690	0	1	485	722	0.9640	0.9636	0.7474	0.0062	0.0069	0.0133	0.0069	0.0070	0.0172	0.0172	0.0048	0.0143	0.0162
399	37.60	689	0	1	486	721	0.9640	0.9636	0.7469	0.0062	0.0069	0.0133	0.0069	0.0070	0.0172	0.0172	0.0048	0.0143	0.0162
556	51.33	486	0	1	670	537	0.9341	0.9337	0.6552	0.0091	0.0107	0.0153	0.0107	0.0107	0.0218	0.0218	0.0064	0.0175	0.0205
557	51.37	485	0	1	671	536	0.9341	0.9337	0.6548	0.0091	0.0107	0.0153	0.0107	0.0107	0.0218	0.0218	0.0063	0.0175	0.0205
558	51.47	484	0	2	673	534	0.9341	0.9337	0.6543	0.0091	0.0107	0.0153	0.0107	0.0108	0.0218	0.0218	0.0063	0.0175	0.0206
559	51.50	482	0	1	674	533	0.9341	0.9337	0.6538	0.0091	0.0107	0.0153	0.0107	0.0108	0.0218	0.0218	0.0063	0.0175	0.0206
560	51.70	481	0	2	676	531	0.9341	0.9337	0.6534	0.0091	0.0107	0.0153	0.0108	0.0108	0.0219	0.0219	0.0063	0.0175	0.0207
696	65.23	306	0	1	841	366	0.9130	0.9127	0.5813	0.0113	0.0152	0.0170	0.0147	0.0148	0.0267	0.0267	0.0072	0.0215	0.0258
697	65.33	305	0	1	842	365	0.9130	0.9127	0.5808	0.0113	0.0152	0.0170	0.0147	0.0148	0.0267	0.0267	0.0072	0.0215	0.0258
698	65.40	304	0	1	843	364	0.9130	0.9127	0.5804	0.0113	0.0152	0.0170	0.0148	0.0148	0.0267	0.0267	0.0072	0.0216	0.0259
699	65.50	303	0	1	844	363	0.9130	0.9127	0.5800	0.0113	0.0152	0.0170	0.0148	0.0148	0.0268	0.0268	0.0072	0.0216	0.0259
700	65.67	302	0	1	845	362	0.9130	0.9127	0.5796	0.0113	0.0152	0.0170	0.0148	0.0148	0.0268	0.0268	0.0072	0.0216	0.0259
904	101.87	65	0	1	1071	136	0.8610	0.8607	0.4701	0.0198	0.0350	0.0223	0.0297	0.0297	0.0409	0.0409	0.0108	0.0424	0.0428
905	102.90	64	0	1	1072	135	0.8610	0.8607	0.4697	0.0198	0.0350	0.0224	0.0298	0.0298	0.0410	0.0410	0.0108	0.0428	0.0430
906	103.10	63	0	1	1073	134	0.8610	0.8607	0.4693	0.0198	0.0350	0.0224	0.0299	0.0299	0.0411	0.0411	0.0108	0.0431	0.0431
907	103.30	62	0	1	1074	133	0.8610	0.8607	0.4690	0.0198	0.0350	0.0225	0.0300	0.0300	0.0413	0.0412	0.0108	0.0434	0.0433
908	103.43	61	0	1	1075	132	0.8610	0.8607	0.4686	0.0198	0.0350	0.0225	0.0301	0.0301	0.0414	0.0414	0.0107	0.0437	0.0434
923	108.90	46	0	1	1090	117	0.8610	0.8607	0.4630	0.0198	0.0350	0.0233	0.0320	0.0320	0.0434	0.0433	0.0106	0.0500	0.0461
924	108.93	45	0	1	1091	116	0.8610	0.8607	0.4626	0.0198	0.0350	0.0234	0.0321	0.0322	0.0435	0.0435	0.0106	0.0506	0.0463
925	109.17	44	0	2	1093	114	0.8610	0.8607	0.4623	0.0198	0.0350	0.0234	0.0324	0.0324	0.0437	0.0437	0.0106	0.0511	0.0467
926	109.87	42	0	1	1094	113	0.8610	0.8607	0.4619	0.0198	0.0350	0.0235	0.0325	0.0326	0.0438	0.0438	0.0106	0.0523	0.0469
927	109.90	41	0	1	1095	112	0.8610	0.8607	0.4615	0.0198	0.0350	0.0236	0.0327	0.0327	0.0440	0.0440	0.0106	0.0529	0.0471
962	129.73	5	0	1	1131	76	0.8610	0.8607	0.4484	0.0198	0.0350	0.0297	0.0397	0.0397	0.0571	0.0571	0.0103	0.1489	0.0570
963	130.23	4	0	1	1132	75	0.8610	0.8607	0.4481	0.0198	0.0350	0.0304	0.0399	0.0400	0.0584	0.0584	0.0103	0.1664	0.0574
964	132.23	3	0	1	1133	74	0.8610	0.8607	0.4477	0.0198	0.0350	0.0313	0.0402	0.0403	0.0601	0.0601	0.0103	0.1921	0.0578
965	132.67	2	0	1	1134	73	0.8610	0.8607	0.4473	0.0198	0.0350	0.0325	0.0405	0.0405	0.0626	0.0626	0.0103	0.2351	0.0582
966	133.80	1	0	1	1135	72	0.8610	0.8607	0.4469	0.0198	0.0350	0.0350	0.0408	0.0408	0.0674	0.0673	0.0103	0.3324	0.0586

In case of a large sample size results change to some extent, our method yields better results than the adjusted hybrid method. The regular method gives a much larger variance than the Peto's, Greenwood's, adjusted, and weighted variance, though decreasing in the end stages, i.e., it underestimates the variance at the end stages. The combination of the Kaplan–Meier and shrunken survival probabilities with the weighted *SE* gives the better results right from start to end. Also, the combination of weighted survival probabilities with the Peto's variance yields better results than the peto's variance based on the Kaplan–Meier survival probabilities. The same is the case with the weighted probabilities in the hybrid variance giving larger values than the hybrid variance based on both Kaplan–Meier and shrunken probabilities.

The performance of $\overset{\wedge }{S_{Km}}$, $\overset{\wedge }{S_{KM*}}$, and $\overset{\wedge }{S_{W}}$ in terms of graphical representation.

To obtain the more detailed results, we assume that the data follow some distribution and draw the graph for the thalassaemia data. Suppose, the data follow an exponential distribution and we draw the graph of the hazard function against time to observe the performance of the various methods.

By definition


(21) $$\begin{array}{l} H\;(t)=-\log S\left(t\right) \end{array}$$

**Figure 1:**
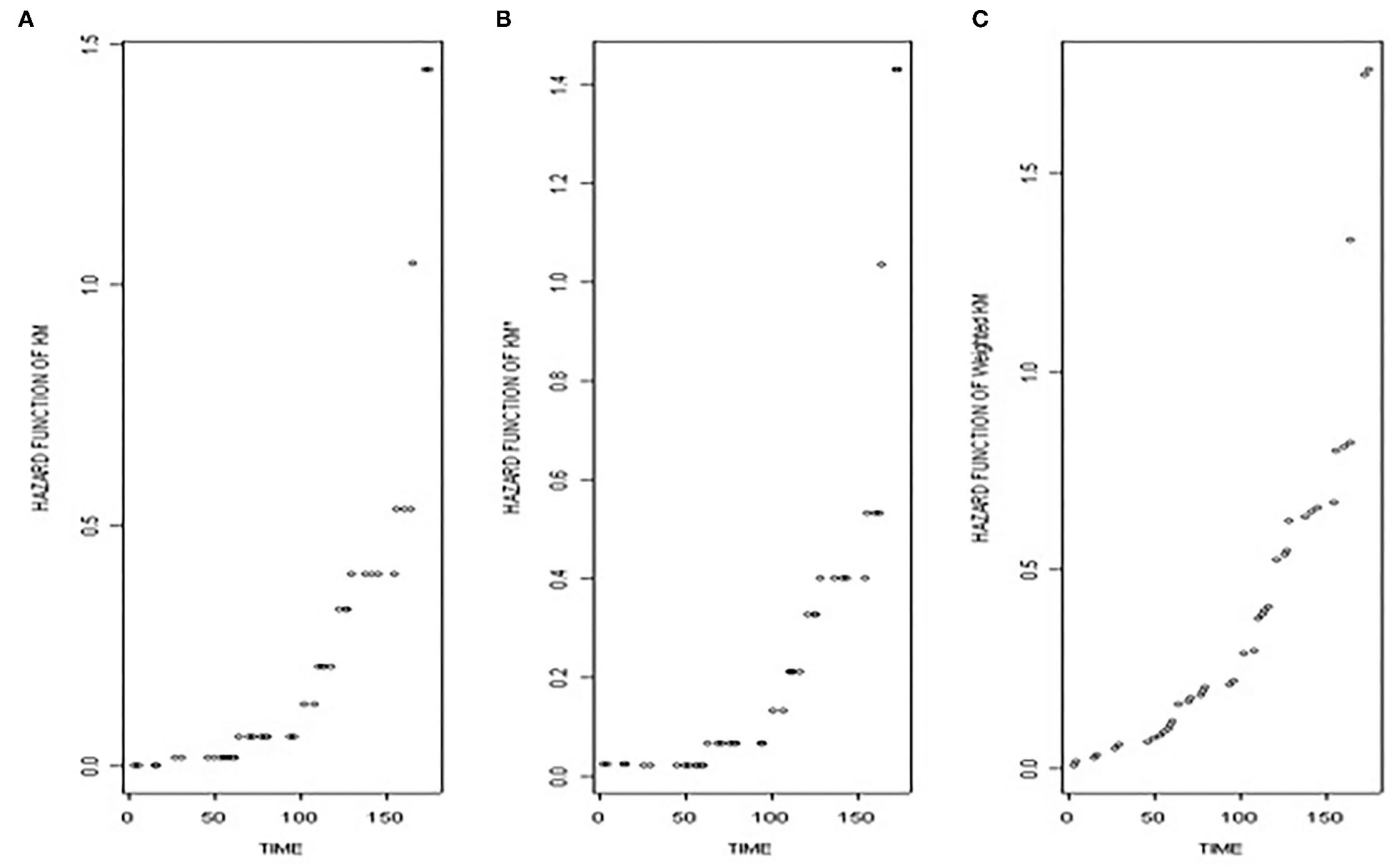
This figure shows the **(A)** the hazard function based on Kaplan–Meier survival function, **(B)** the hazard function based on the shrunken Kaplan–Meier survival function, and **(C)** the hazard function based on the weighted Kaplan–Meier survival function against time.

Here, we assume that the data follow an exponential distribution and plot the three functions, i.e., $\overset{\wedge }{S_{Km}}$, $\overset{\wedge }{S_{KM*}}$, and$\overset{\wedge }{S_{W}}$ against time. Looking at the graph, we see that Figures 1A,B are smooth in the start, as the starting value of Kaplan–Meier survival function is 1 and that of hazard function is zero. In contrast, the starting value of shrunken function is not 1, so its starting point (in case if the initial value is censored) can never be zero. Nevertheless, it remains constant in the start, as it does not give any significance to the censored observations, only changing when an event occurred. Looking at the third figure, though, which is unlike the first two, more detailed information is given not only in the start, but also at each stage as it changes with the event as well as with the censoring.

## Discussion

The Kaplan–Meier product limit estimator has become an important tool in the analysis of censored survival data. It is easy to compute and understand. For the variance of the Kaplan–Meier, Greenwood's and Peto's estimators are used, in case of low censoring, these methods give good results although, in case of moderate or heavy censoring, though they underestimate the true variance in the tails. To overcome these deficiencies, Borkowf proposed the regular and adjusted hybrid variance estimators for the Kaplan–Meier survival function. These methods also give the variances in those times where no event occurred. But for a small sample, in most of the cases, they give the larger variance and also adjusted hybrid method gives a variance greater than the binomial variance in case of no censoring. Moreover, in cases of relatively large sample sizes, it gives the smaller variance than the standard Greenwood's and Peto's variance on the right tail.

To overcome these difficulties, a weighted Kaplan–Meier survival function and its variance are proposed, which unlike the Kaplan–Meier and shrunken Kaplan–Meier functions gives the estimate of each point as well as the variance. It possesses all the characteristics of Kaplan–Meier and in case of no censoring, it is equal to Kaplan–Meier and has a binomial variance.

In case of very heavy censoring or complete censoring, the new method performs better than the others. In the latter case, adjusted hybrid variance is unable to provide the variance of the last censored observation, while the new method is able to provide it, along with the survival probabilities at each point.

In case small sample size with moderate censoring, the performance of the weighted variance is the same as that of the regular and adjusted hybrid variances, while in case of large sample size and heavy censoring it performs better than the others.

If the initial times are censored then the weighted method describes the pattern of the curve in more detail compared to the curves based on the Kaplan–Meier survival function and shrunken Kaplan–Meier function, respectively.

If the dataset does not contain any censoring time then the weighted Kaplan–Meier and weighted variance reduced to the complement of empirical distribution and the variance becomes simply the binomial variance, as in the traditional method. Nonetheless, the variance of adjusted hybrid variance remains larger than the others.

The performance of Peto is also improved by using the weighted Kaplan–Meier survival function, while considering it in the Greenwood formula does not improve the results. In contrast, taking the Kaplan–Meier survival function and shrunken Kaplan–Meier function and combining them with the weighted variance, they perform much better in all situations. This combination behaves according to the situation, whether there is no censoring, moderate censoring, or heavy censoring. Moreover, it is suitable for all sample sizes.

In summary, the weighted Kaplan–Meier estimator gives more detailed information about the pattern of the data in case of low, moderate, and heavy censoring and also if the initial observations are censored. In case of small samples, the weighted, regular, and adjusted hybrid variances can be used depending on the circumstances and the pattern of censoring. In any situation whether there is no, low, moderate, or heavy censoring, the adjusted hybrid method gives a larger variance than the Greenwood's and Peto's for a very small sample. This is most marked on the left tail in case of censoring. When there is no censoring, though, the variance is always greater than the binomial variance. In contrast to this, the weighted method behaves accordingly to the situation.

## Data Availability Statement

The raw data supporting the conclusions of this article will be made available by the authors, without undue reservation.

## Author Contributions

All authors listed have made a substantial, direct, and intellectual contribution to the work and approved it for publication.

## Conflict of Interest

The authors declare that the research was conducted in the absence of any commercial or financial relationships that could be construed as a potential conflict of interest.

## Publisher's Note

All claims expressed in this article are solely those of the authors and do not necessarily represent those of their affiliated organizations, or those of the publisher, the editors and the reviewers. Any product that may be evaluated in this article, or claim that may be made by its manufacturer, is not guaranteed or endorsed by the publisher.
