# Supplementary material for: Methods for Improving the Variance Estimator of the Kaplan–Meier Survival Function, When There Is No, Moderate and Heavy Censoring-Applied in Oncological Datasets
Source: Front Public Health. 2022 May 26;10:793648. doi: 10.3389/fpubh.2022.793648 (PMC9178555; doi:10.3389/fpubh.2022.793648)
Supplement: Supplementary file 1 [file Data_Sheet_1.docx]

# Appendix

# Variance of the weighted Kaplan–Meier estimater

The weight is

$$W_{i}=\left(1-\frac{\left(k*I_{ci}\right)}{\left(n*r_{i}\right)}\right)$$

The modified Kaplan–Meier estimate of the survival function for any value of *t* in the interval *t*_(i)_ to *t*_(i+1)_ can be written as

Where $p_{i}^{*}=\left(\frac{n_{i}-d_{i}}{n_{i}}\right)*\left(1-\frac{k*I_{ci}}{n*r_{i}}\right)$ is the estimated probability that an individual survives through the time interval which begins at *t*_(i)_, *i* = 1,2,…,n

Taking logarithms,

and so the variance of $\log\mathop{S_{w}\left(i\right)}\limits$ is given by

The number of individuals who survive through the interval beginning at *t*_(i)_ can be assumed to have a binomial distribution with parameters *r_i_* and *p_i_*, where *p_i_* is the true probability of survival through that interval. The observed number who survived is $\left(r_{i}-d_{i}\right)$.

Therefore,

$$\mathrm{var}\left(r_{i}-d_{i}\right)=r_{i}p_{i}\left(1-p_{i}\right)$$

Since

$$p_{i}^{*}=\left\{\frac{\left(r_{i}-d_{i}\right)}{r_{i}}*\left(1-\frac{k*I_{ci}}{n*r_{i}}\right)\right.$$

Therefore,

$$\begin{matrix} Var\left(\frac{\left(r_{i}-d_{i}\right)}{r_{i}}*\left(1-\frac{k*I_{ci}}{n*r_{i}}\right)\right)=\frac{p_{i}\left(1-p_{i}\right)}{r_{i}}\left(1-\frac{k*I_{ci}}{n*r_{i}}\right)^{2} \\ \end{matrix}$$

so the variance of $p_{i}^{*}$ may then be estimated by

$$\begin{matrix} Var\left(p_{i}^{*}\right)=\frac{p_{i}^{*}\left(1-p_{i}^{*}\right)}{r_{i}}\left(1-\frac{k*I_{ci}}{n*r_{i}}\right)^{2} \\ \end{matrix}$$

In order to obtain the variance of $\log p_{i}^{*}$, we make use of a general result for the approximate variance of a function of a random variable by Taylor’s series approximation.

$$\mathrm{var}\left\{g\left(t\right)\right\}\approx\left\{\frac{dg\left(t\right)}{dt}\right\}^{2}\mathrm{var}\left(t\right)$$

Using the above equation,

so

$$\mathrm{var}\left\{\log p_{i}^{*}\right\}=$$

$$\begin{matrix} \frac{p_{i}^{*}\left(1-p_{i}^{*}\right)}{r_{i}}\left(1-\frac{k*I_{ci}}{n*r_{i}}\right)^{2} \\ \end{matrix}$$

Which on substitution for $p_{i}^{*}$, reduces to

$$\mathrm{var}\left\{\log p_{i}^{*}\right\}=\frac{\left(k*I_{ci}\left(r_{i}-d_{i}\right)+n*r_{i}*d_{i}\right)}{r_{i}^{2}n\left(r_{i}-d_{i}\right)}\left(1-\frac{k*I_{ci}}{n*r_{i}}\right)$$

So

By using the Taylor’s approximation again to find the

and so

$$\frac{\left(k*I_{ci}\left(r_{i}-d_{i}\right)+n*r_{i}*d_{i}\right)}{n_{i}^{2}N\left(n_{i}-d_{i}\right)}\left(1-\frac{k*I_{ci}}{n*r_{i}}\right)$$

and the *SE*

Required formula
